# Correction to: A systematic review of population and patient perspectives and experiences as measured in Latin American and Caribbean surveys

**DOI:** 10.1093/heapol/czad104

**Published:** 2023-11-06

This is a correction to: Jesús Medina-Ranilla, Laura Espinoza-Pajuelo, Agustina Mazzoni, Javier Roberti, Ezequiel García—Elorrio, Hannah Hogan Leslie, Patricia Jannet García, A systematic review of population and patient perspectives and experiences as measured in Latin American and Caribbean surveys, *Health Policy and Planning*, 2023; czad083, https://doi.org/10.1093/heapol/czad083

A change has been made to the author list.

